# Association between Hashimoto’s Thyroiditis and Thyroid Cancer in 64,628 Patients

**DOI:** 10.3389/fonc.2017.00053

**Published:** 2017-04-10

**Authors:** Christina Resende de Paiva, Christian Grønhøj, Ulla Feldt-Rasmussen, Christian von Buchwald

**Affiliations:** ^1^Department of Otorhinolaryngology, Head and Neck Surgery and Audiology, Rigshospitalet, University of Copenhagen, Copenhagen, Denmark; ^2^Department of Medical Endocrinology, Rigshospitalet, University of Copenhagen, Copenhagen, Denmark

**Keywords:** Hashimoto’s thyroiditis, autoimmune thyroid disease, thyroid cancer, autoimmune thyroiditis, Hashimoto’s disease

## Abstract

**Background:**

The incidence of thyroid cancer (TC) is increasing although explanatory causes are lacking. A link between cancer and inflammation is well documented but unclear for autoimmune thyroid diseases and TC. We aimed to systematically review the association between Hashimoto’s thyroiditis (HT) and papillary, follicular, medullary, anaplastic thyroid carcinoma, and thyroid lymphoma (TL).

**Methods:**

PubMed, OVID Medline, Google Scholar, and the Cochrane Library were searched from 1955 to 2016. The inclusion criteria were age >18 years, ≥20 cases of HT or TC. We collectively examined the incidence of HT in TC and of TC in HT.

**Results:**

We identified 36 studies (64,628 subjects) published between 1955 and 2016 from 13 countries. We found a relative risk (RR) of HT among papillary thyroid cancer (PTC) of 2.36 [95% confidence intervals (CIs) 1.55–3.29, *p* < 0.001], an RR of PTC among HT of 1.40 (95% CI 1.07–1.85, *p* = 0.016), and an RR of TL among HT of 9.74 (95% CI 3.93–24.13, *p* < 0.001).

**Conclusion:**

We report an association between HT and PTC and between HT and TL. No association was found between HT and follicular, medullary, or anaplastic thyroid cancer.

## Introduction

Thyroid cancer (TC) is the most common endocrine malignancy, and the incidence has been increasing worldwide during the last decades (1–5). Papillary thyroid carcinoma (PTC) (70–80% of all TCs), follicular thyroid carcinoma (FTC) (10–20% of all TCs), medullary thyroid carcinoma (MTC) (5–8% of all TCs), and anaplastic thyroid carcinoma (ATC) (<5% of all TCs) collectively comprise more than 98% of all thyroid malignancies (6–8). PTC and FTC are well-differentiated TCs and generally have a favorable prognosis (7), contrary to MTC and ATC with poor prognosis. Thyroid lymphoma (TL) is a rare TC, accounting for 1–5% of all thyroid malignancies (9).

Hashimoto’s thyroiditis (HT), e.g., chronic lymphocytic or autoimmune thyroiditis, is a chronic inflammation of the thyroid gland, the most common inflammatory thyroid disease, and the typical cause of hypothyroidism (8, 10, 11). Several diagnostic methods are used to define HT including ultrasonography-guided fine needle aspiration cytology (FNAC), thyroid autoantibodies, and surgical material (12), and studies correspondingly include HT patients based on different criteria posing a challenge for comparison.

A link between cancer and inflammation is well recognized. As early as 1863, Rudolf Virchow noted leukocytes in neoplastic tissue and suggested a correlation to the development of cancer (13, 14). The association between HT and PTC was first described by Dailey et al. in 1955 (15), and since then, conflicting results have been reported regarding the association between HT and thyroid malignancies.

Few systematic reports are published examining the association between HT and TC, all investigating PTC, and all fail to distinguish HT patients from TC patients, which complicate comparisons (11, 16, 17). A systematic review and meta-analysis examining the association between HT and TCs other than PTC and examining the incidences of HT in TC and of TC in HT has not been published.

The aim of this systematic review was to examine the association between HT and TC.

## Materials and Methods

### Systematic Literature Search and Eligibility Criteria

This systematic review and meta-analysis was conducted with reference to the Preferred Reporting Items for Systematic Reviews and Meta-Analysis statement (18).

In August 2016, one author (Christina Resende de Paiva) systematically searched PubMed, OVID Medline, Google Scholar, and the Cochrane Library. The search strategy was carried out using the keywords and MESH terms “Hashimoto Disease” OR “Hashimoto Thyroiditis” OR “Chronic Lymphocytic Thyroiditis” AND “Thyroid Neoplasms” OR “Thyroid Cancer” OR “Thyroid Malignancy.”

We included both retrospective and prospective studies that examined the relationship between HT and any of the following TCs: PTC, FTC, MTC, ATC, and TL. The inclusion criteria were restricted to English or Portuguese language, published between January 1, 1955 and August 30, 2016, age >18 years and ≥20 cases of HT or TC.

We excluded case studies, review articles, articles only providing data about TC in general as opposed to a subtype of TC, and articles that did not include data about all cancer cases (i.e., only examining microcarcinomas).

### Data Extraction, Synthesis, and Statistical Analysis

The following information was recorded by one author (Christina Resende de Paiva): author, gender distribution, age, year of publication, country, study design, methods of diagnosis, sample size, type of cancer, and number of cancer and HT cases. Included studies were categorized into two groups: one group examining HT in TC and another examining TC in HT.

Statistics were carried out using MedCalc statistical software version 16.8.4. For analysis, we calculated the relative risks (RRs), the 95% confidence intervals (CIs), and the pooled effects. A *p* value below 0.05 was considered significant.

## Results

**Figure 1:**
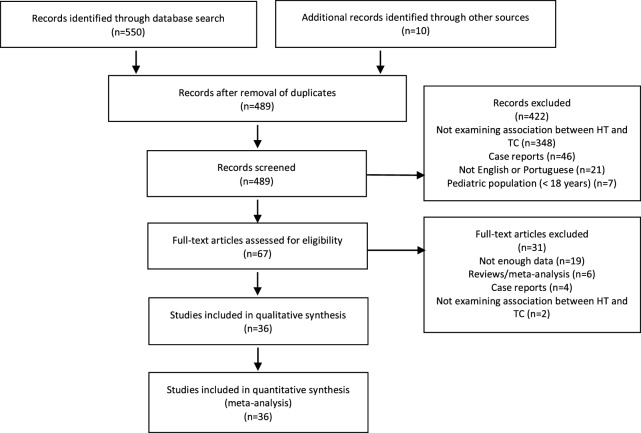
**Preferred Reporting Items for Systematic Reviews and Meta-Analysis flow diagram of article selection**. HT, Hashimoto’s thyroiditis; TC, thyroid cancer.

The literature search yielded a total of 550 records. From these, we manually selected 57 articles for full-text assessment, of which 31 articles were excluded. Accordingly, 26 studies were left eligible for inclusion, and additional 10 studies were identified through reference lists yielding a total of 36 records (*n* = 64,628 subjects) (Figure 1; Tables 2 and 3). Nineteen studies (*n* = 23,848) examined HT among TC (Table 2), and 19 studies (*n* = 41,643) examined TC among HT (Table 3). Of these, 863 patients were included in both analyses (19).

In the pooled analysis, most patients were females (*n* = 8,828, 82.1%). The median age among patients with TC and HT was 45.9 years while 47 years among patients with TC without HT. Seventeen studies (*n* = 37,556 subjects) were European, Canadian, or US based, 15 studies (*n* = 25,261 subjects) were Asian, and 4 studies (*n* = 1,811 subjects) were Brazilian.

### HT in TC

The HT in TC group included 19 studies (*n* = 23,848) published between 1998 and 2016. Eight studies had a group of patients useful as control group enabling us to calculate a RR while 11 studies did not supply data for this purpose. All studies in this group examined material from thyroidectomies (Table 2).

In the pooled analysis, most patients were women (*n* = 8,050, 82.4%). The median age for patients with TC and HT was 45.1 years while it was 46.7 years among patients with TC without HT. Four studies (*n* = 787 subjects) were European, Canadian, or US based, 11 studies (*n* = 21,250 subjects) were Asian, and 4 studies (*n* = 1,811 subjects) were Brazilian.

#### HT in PTC

**Figure 2:**
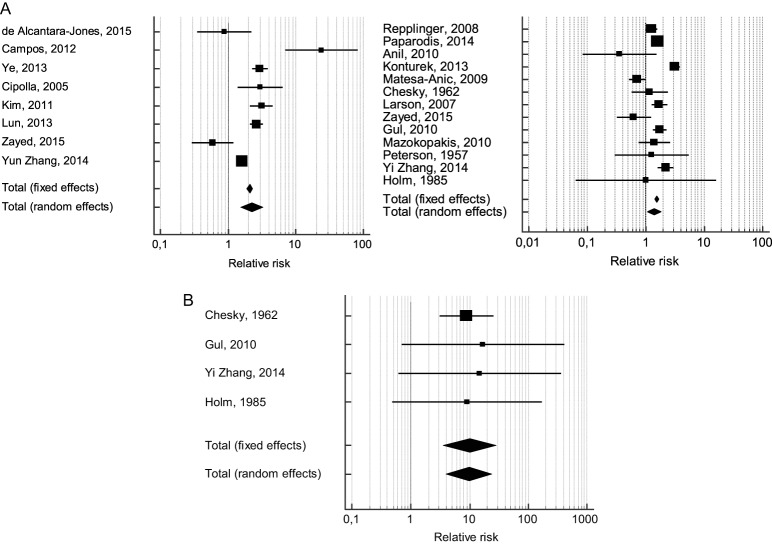
**Relative risk (RR) of Hashimoto’s thyroiditis (HT) in thyroid cancers (TCs) and of TCs in HT**. **(A)** RR of HT in patients with papillary thyroid cancer (PTC) (left) and of PTC in patients with HT (right). **(B)** RR of thyroid lymphoma in patients with HT.

Among patients with PTC (*n* = 11,155), 18.90% had HT (*n* = 2,108) (16, 19–29, 31–36). The female:male (F:M) ratio was 4.8:1 (7,815:1,623) for PTC patients, and the RR of having HT in PTC patients varied from 0.60 (95% CI 0.29–1.21) to 23.97 (95% CI 6.98–82.29). With 915 HT cases among 4,725 PTC patients and a control group of 894 HT cases among 10,862 patients without cancer (non-TC), the pooled effect of the RRs was 2.26 (95% CI 1.55–3.29, *p* < 0.001) (Figure 2).

An association was found between HT and PTC.

#### HT in FTC

Among patients with FTC (*n* = 363), 4.68% had HT (*n* = 17) (16, 19, 25, 27, 29). The F:M ratio was 2.5:1 (226:89) for FTC patients, and the RR of having HT in FTC patients varied from 0.18 (95% CI 0.012–2.85) to 1.0 (95% CI 0.40–2.49). With 5 HT cases among 79 FTC patients and a control group of 91 HT cases among 932 non-TC patients, the pooled effect of the RRs was 0.66 (95% CI 0.14–3.13, *p* = 0.599).

No association was found between HT and FTC.

#### HT in MTC

One study (*n* = 863 subjects) examined the association between HT and MTC (19). Among patients with MTC (*n* = 15), 20% had HT (*n* = 3) with a RR of 2.04 (95% CI 0.72–5.75). The F:M ratio was 1.5:1 (9:6).

No association was found between HT and MTC.

#### HT in ATC

No studies examined this association.

#### HT in TL

Two studies examined the association between HT and TL, but none of them had a control group of HT cases among non-TC patients (16, 30). Therefore, the RR could not be calculated. Nevertheless, 35.14% of patients with TL (*n* = 37) had HT (*n* = 13). There were no data on the F:M ratio.

### TC in HT

The TC in HT group included 19 studies (*n* = 41,643) published between 1955 and 2015. Thirteen studies had a control group, and six studies did not supply data for this purpose. Fifteen studies examined material from thyroidectomies while four studies examined FNAC material (Table 3).

In the pooled analysis, most patients were women (*n* = 778, 78.7%). The median age among patients with TC and HT was 46.6 and 47.2 years among patients with TC without HT. Five studies (*n* = 19,898 subjects) were European, nine studies (*n* = 16,871 subjects) were US based, and four studies (*n* = 4,874 subjects) were Asian.

#### PTC in HT

Among patients with HT (*n* = 7,873), 9.03% had PTC (*n* = 711) (15, 19, 24, 37–52). Six studies reported the F:M ratio among patients with PTC to be 3.9:1 (732:190). The RR of having PTC in HT patients varied from 0.35 (95% CI 0.08–1.50) to 3.1 (95% CI 2.61–3.78). With 601 PTC cases among 5,312 HT patients and a control group of 2,236 PTC cases among 31,708 non-HT patients, the pooled effect of the RRs was 1.40 (95% CI 1.07–1.85, *p* = 0.016) (Figure 2).

An association was found between PTC and HT.

#### FTC in HT

Among patients with HT (*n* = 2,296), 1.26% had FTC (*n* = 29) (19, 38, 43–45, 49, 51, 52). Two studies reported the F:M ratio among patients with FTC to be 2.4:1 (34:14). The RR of having FTC in HT patients varied from 0.18 (95% CI 0.01–2.94) to 5.43 (95% CI 2.35–12.56). With 25 FTC cases among 2,204 HT patients and a control group of 267 FTC cases among 13,953 non-HT patients, the pooled effect of the RRs was 0.95 (95% CI 0.35–2.57, *p* = 0.915).

No association was found between FTC and HT.

#### MTC in HT

Among patients with HT (*n* = 370), 1.62% had MTC (*n* = 6) (19, 45, 49, 51). One study reported the F:M ratio among patients with MTC as 1.5:1 (9:6). The RR of having MTC in HT patients varied from 0.51 (95% CI 0.029–9.15) to 4.99 (95% CI 0.71–35.05). With 5 MTC cases among 278 HT patients and a control group of 19 MTC cases among 1,845 non-HT patients, the poled effect of the RRs was 2.49 (95% CI 0.93–6.67, *p* = 0.07).

No association was found between MTC and HT.

#### ATC in HT

Among patients with HT (*n* = 206), 0.49% had ATC (*n* = 1) (44, 51). One study reported the F:M ratio among patients with ATC as 3:0. The RR of having ATC in HT patients varied from 0.71 (95% CI 0.04–13.60) to 3.62 (95% CI 0.33–39.58). With one ATC case among 206 HT patients and a control group of five ATC cases among 1,249 non-HT patients, the pooled effect of the RRs was 1.90 (95% CI 0.30–12.19, *p* = 0.499).

No association was found between ATC and HT.

#### TL in HT

Among patients with HT (*n* = 3,497), 0.37% had TL (*n* = 13) (41, 43, 45, 51, 52). No studies reported the F:M ratio among patients with TL. The RR of having TL in HT patients varied from 8.86 (95% CI 3.09–25.38) to 16.84 (95% CI 0.69–410.24). With 11 cases of TL among 1,461 HT patients and a control group of 11 cases of TL among 10,307 non-HT patients, the pooled effect of the RRs was 9.74 (95% CI 3.93–24.13, *p* < 0.001) (Figure 2).

An association was found between TL and HT.

We observe an association between HT and PTC among both patient groups and a corresponding association between HT and TL among HT patients. No association was found between HT and FTC or MTC in both groups of patients neither an association between HT and ATC among HT patients (Table 1). The remaining associations could not be evaluated due to lack of data. In all subtypes of TC, females were more often affected than males with F:M ratios ranging from 1.5:1 to 4.8:1.

**Table 1:** **Overview of the associations between HT and TC**.

		PTC	FTC	MTC	ATC	TL
HT in TC group	HT	Associated (*p* < 0.001)	Not associated (*p* = 0.599)	Not associated (*p* = 0.18)	N/A	N/A
TC in HT group	HT	Associated (*p* = 0.016)	Not associated (*p* = 0.915)	Not associated (*p* = 0.07)	Not associated (*p* = 0.499)	Associated (*p* < 0.001)

*N/A, not available; HT, Hashimoto’s thyroiditis; TC, thyroid cancer; PTC, papillary thyroid cancer; FTC, follicular thyroid cancer; MTC, medullary thyroid cancer; ATC, anaplastic thyroid cancer; TL, thyroid lymphoma*.

**Table 2:** **Studies examining the incidence of HT in TC**.

Reference	Country	Study type	Study method	Sample size	Type of cancer	TC cases (*n*)	HT cases (*n*)	TC cases (F:M)	TC + HT (age, median)	TC ÷ HT (age, median)	% HT in TC (HT/TC)	% HT in non-TC (HT/non-TC) (control group)	Relative risk	95% CI
(20)	Brazil	Retrospective	Thyroidectomy	49	Papillary	33	14	30:3	48.5	43.2	27.27 (9/33)	31.25 (5/16)	0.87	0.35–2.18
(21)	China	Retrospective	Thyroidectomy	619	Papillary	619	222	484:135	45.9	45.5	35.86 (222/619)	N/A	N/A	N/A
(22)	Brazil	Retrospective	Thyroidectomy	315	Papillary	41	14	34:7	44.9	49.1	26.83 (11/41)	1.12 (3/268)	23.97	6.98–82.29
(23)	China	Retrospective	Thyroidectomy	2,052	Papillary	1,004	254	828:176	N/A	N/A	18.63 (187/1,004)	6.42 (66/1,028)	2.90	2.22–3.79
(24)	Italy	Retrospective	Thyroidectomy	178	Papillary	71	27	68:21	N/A	N/A	26.76 (19/71)	8.99 (8/89)	2.98	1.39–6.40
(25)	Korea	Retrospective	Thyroidectomy	1,329	Papillary	1,028	336	821:207	47.5	48	29.86 (307/1,028)	9.64 (24/249)	3.10	2.09–4.58
					Follicular	52		32:20			9.62 (5/52)		1.00	0.40–2.49
(26)	Korea	Retrospective	Thyroidectomy	303	Papillary	269	60	225:44	42.8	48.3	21.56 (58/269)	N/A	N/A	N/A
(27)	China	Retrospective	Thyroidectomy	1,997	Papillary	1,788	93	1,450:338	39.9	40.8	4.75 (85/1,788)	N/A	N/A	N/A
					Follicular	209		153:56			3.83 (8/209)			
(28)	China	Retrospective	Thyroidectomy	2,478	Papillary	676	256	538:138	41.3	44.8	18.79 (127/676)	7.16 (129/1,802)	2.624	2.09–3.30
(29)	Brazil	Retrospective	Thyroidectomy	1,395	Papillary	93	74	81:12	N/A	N/A	18.28 (17/93)	N/A	N/A	N/A
					Follicular	27		23:4			11.11 (3/27)			
(19)	Jordan	Retrospective	Thyroidectomy	863	Medullary	15	78	9:6	51.3	49.9	20 (3/15)	9.81 (67/683)	2.04	0.72–5.75
					Papillary	137		102:35			5.84 (8/137)		0.60	0.29–1.21
					Follicular	27		18:9			0 (0/27)		0.18	0.01–2.85
(30)	Canada	Retrospective	Thyroidectomy	20	Lymphoma	20	12	N/A	N/A	N/A	60 (12/20)	N/A	N/A	N/A
(16)	USA	Retrospective	Thyroidectomy	453	Papillary	388	59	267:121	41	43	14.69 (57/388)	N/A	N/A	N/A
					Follicular	48		N/A			2.08 (1/48)			
					Lymphoma	17		N/A			5.88 (1/17)			
(31)	China	Retrospective	Thyroidectomy	8,524	Papillary	1,735	839	N/A	43.1	46.6	14.24 (247/1,735)	8.80 (592/6,727)	1.62	1.41–1.86
(32)	Brazil	Retrospective	Thyroidectomy	52	Papillary	52	17	48:4	51.3	43.1	32.69 (17/52)	N/A	N/A	N/A
(33)	Korea	Retrospective	Thyroidectomy	1,357	Papillary	1,357	359	1,176:181	44.5	45.9	26.46 (359/1,357)	N/A	N/A	N/A
(34)	Japan	Retrospective	Thyroidectomy	1,533	Papillary	1,533	281	1,402:131	42.6	48.6	18.33 (281/1,533)	N/A	N/A	N/A
(35)	USA	Retrospective	Thyroidectomy	136	Papillary	136	41	95:41	45.5	54	30.15 (41/136)	N/A	N/A	N/A
(36)	Korea	Retrospective	Thyroidectomy	195	Papillary	195	56	166:29	45.9	49.6	28.72 (56/195)	N/A	N/A	N/A

*N/A, not available; TC, thyroid cancer; HT, Hashimoto’s thyroiditis; F, female; M, male; CI, confidence interval*.

**Table 3:** **Studies examining the incidence of TC in HT**.

Reference	Country	Study type	Study method	Sample size	Type of cancer	TC cases (*n*)	HT cases (*n*)	TC cases (F:M)	TC + HT (age, median)	TC ÷ HT (age, median)	% TC in HT (TC/HT)	% TC in non-HT (TC/non-HT) (control group)	Relative risk	95% CI
(37)	USA	Retrospective	Thyroidectomy	1,198	Papillary	292	217	215:77	N/A	N/A	29.03 (63/217)	23.34 (229/981)	1.24	0.98–1.58

(38)	USA	Retrospective	Thyroidectomy	2,718	Papillary	807	567	N/A	N/A	N/A	42.68 (242/567)	26.27 (565/2,151)	1.63	1.44–1.83
					Follicular	56					1.76 (10/567)	2.14 (46/2,151)	0.83	0.42–1.62

(39)	Turkey	Prospective	Fine needle aspiration cytology (FNAC)	715	Papillary	21	164	N/A	N/A	N/A	1.22 (2/164)	3.45 (19/551)	0.35	0.08–1.50

(24)	Italy	Retrospective	Thyroidectomy	47	Papillary	13	47	12:1	45.7	N/A	27.66 (13/47)	N/A	N/A	N/A

(40)	Poland	Retrospective	Thyroidectomy	7,545	Papillary	636	452	N/A	53.5	52.3	23.45 (106/452)	7.47 (530/7,093)	3.14	2.61–3.78

(41)	Japan	Retrospective	FNAC	2,036	Papillary	36	2,036	N/A	N/A	N/A	1.77 (36/2,036)	N/A	N/A	N/A
					Lymphoma	2					0.10 (2/2,036)			

(42)	Croatia	Retrospective	FNAC	10,508	Papillary	269	2,156	236:33	50	51	1.95 (42/2,156)	2.72 (227/8,352)	0.72	0.58–0.99

(15)	USA	Prospective	Thyroidectomy	2,336	Papillary	29	278	N/A	37.5	36	10.43 (29/278)	N/A	N/A	N/A

(43)	USA	Retrospective	Thyroidectomy	8,850	Papillary	141	432	N/A	N/A	N/A	1.85 (8/432)	1.58 (133/8,418)	1.17	0.58–2.38
					Follicular	173					0.93 (4/432)	2.01 (169/8,418)	0.46	0.17–1.24
					Lymphoma	16					1.16 (5/432)	0.13 (11/8,418)	8.86	3.09–25.38

(44)	USA	Retrospective	Thyroidectomy	812	Papillary	179	98	142:37	41	39	34.7 (34/98)	20.4 (145/710)	1.699	1.25–2.31
					Follicular	21		16:5			9.2 (9/98)	1.69 (12/710)	5.434	2.35–12.56
					Anaplastic	3		3:0			1.02 (1/98)	0.28 (2/710)	3.622	0.33–39.58

(19)	Jordan	Retrospective	Thyroidectomy	863	Medullary	15	78	9:6	51.3	49.9	3.85 (3/78)	1.53 (12/785)	2.516	0.73–8.73
					Papillary	137		102:35			10.26 (8/78)	16.43 (129/785)	0.624	0.32–1.23
					Follicular	27		18:9			0 (0/78)	3.44 (27/785)	0.181	0.01–2.94

(45)	Turkey	Retrospective	Thyroidectomy	613	Papillary	171	92	N/A	43	46.5	43.48 (40/92)	25.14 (131/521)	1.729	1.31–2.28
					Follicular	11		N/A			1.09 (1/92)	1.92 (10/521)	0.566	0.07–4.37
					Medullary	5		N/A			0 (0/92)	0.96 (5/521)	0.510	0.03–9.15
					Lymphoma	1		N/A			1.09 (1/92)	0 (0/521)	16.839	0.69–410.24

(46)	Greece	Retrospective	Thyroidectomy	140	Papillary	32	42	25:7	49.3	54.7	28.57 (12/42)	20.41 (20/98)	1.400	0.76–2.60

(47)	USA	Retrospective	Thyroidectomy	48	Papillary	6	48	N/A	51.7	N/A	12.5 (6/48)	N/A	N/A	N/A

(48)	USA	Retrospective	Thyroidectomy	60	Papillary	2	60	N/A	N/A	N/A	3.33 (2/60)	N/A	N/A	N/A

(49)	USA	Retrospective	Thyroidectomy	92	Papillary	24	92	N/A	N/A	N/A	26.09 (24/92)	N/A	N/A	N/A
					Follicular	4					4.35 (4/92)			
					Medullary	1					1.09 (1/92)			

(50)	USA	Retrospective	Thyroidectomy	757	Papillary	16	77	N/A	N/A	N/A	2.60 (2/77)	2.06 (14/680)	1.262	0.29–5.45

(51)	China	Retrospective	Thyroidectomy	647	Papillary	134	108	N/A	43.3	48.3	37.96 (41/108)	17.25 (93/539)	2.20	1.62–2.98
					Follicular	2		N/A			0 (0/108)	0.37 (2/539)	0.99	0.05–20.50
					Medullary	4		N/A			1.85 (2/108)	0.37 (2/539)	4.99	0.71–35.05
					Anaplastic	3		N/A			0 (0/108)	0.56 (3/539)	0.71	0.04–13.60
					Lymphoma	1		N/A			0.93 (1/108)	0 (0/539)	14.86	0.61–362.45

(52)	Sweden	Prospective	FNA	1,658	Papillary	2	829	N/A	N/A	N/A	0.12 (1/829)	0.12 (1/829)	1.00	0.06–15.96
					Follicular	2					0.12 (1/829)	0.12 (1/829)	1.00	0.06–15.96
					Lymphoma	4					0.48 (4/829)	0 (0/829)	9.00	0.49–166.91

*N/A, not available; TC, thyroid cancer; HT, Hashimoto’s thyroiditis; F, female; M, male; CI, confidence interval*.

## Discussion

To our knowledge, we present the first systematic review on the association between HT and five types of TC, and the largest review on HT and PTC examining both HT and TC patient groups. Based on 64,628 subjects, we report a correlation between HT and PTC among both HT and TC patients and between HT and TL among HT patients. We found no association between HT and FTC or MTC among these patient groups and no association between HT and ATC among HT patients.

The annual incidence of HT is estimated to be 0.3–1.5 cases per 1,000 persons worldwide affecting up to 2% of any general population (53). Thus, our findings impact a large fraction of the global population, and clinicians should consider the higher risk of TC in patients with HT and might consider wider indications for a workup when this patient group presents suspect symptoms of cancer.

It has been proposed that PTC in the setting of HT is associated with a better prognosis, which might be explained by earlier discovery since most HT patients receive more frequent medical checkups for their hypothyroidism (27, 34, 54).

The incidence of TC is increasing, primarily due to papillary microcarcinomas (≤10 mm) (55, 56) possibly due to changes in immigration, diagnostic criteria or surgical interventions of benign thyroid disease as well as increased diagnostic activity, improved diagnostic tools and coincidentally identified microcarcinomas (1). Some countries have introduced iodized salt programs and have since observed increasing trends of TC (1, 57–62), while other countries have not introduced these programs or observed a decrease in iodine intake although the incidence of TC continues to increase here as well (3, 63).

Malignant transformation in the thyroid gland might be caused by cellular mediators produced by immune cells in states of chronic inflammation (14), or by elevated levels of TSH that stimulate follicular epithelial proliferation (64). McLeod et al. conducted a systematic review reporting that elevated TSH levels are associated with an increased risk of TC (65). Similar studies have also reported that elevated TSH values are associated with a more advanced stage of TC (66–69) and that treatment with l-T_4_, by reducing TSH levels, decreases the risk of TC (70). However, these findings are almost exclusively based on patients with PTC and might not be applicable to other subtypes of TC.

Biomolecular markers have been identified as possibly being involved in neoplastic transformation from HT to TC. These include RET/PTC rearrangements, p63 protein expression, BRAF mutation, and PI3K/Akt expression (71–73). For instance, p63 is commonly expressed in HT and in PTC where as there is no such expression in normal thyroid tissue (71). Again, such mechanisms have almost exclusively been investigated in PTC. Thus, we know very little about other subtypes of TC and what might induce their development.

Studies examining the relationship between HT and TC through FNAC tend not to show a higher risk of TC (11). This raises the question of the sensitivity of FNAC and whether FNAC is adequate for monitoring HT. On the other hand, thyroidectomy might identify subclinical TCs that are unlikely to impact overall survival. It has been reported that the chance of finding an incidental papillary carcinoma at autopsy ranges from 3 to 36% (74). Either way, the diagnostic method seems to affect whether an association between HT and TC is found or not and this fact should be considered since most studies included in our analysis are based on thyroidectomies.

Very few studies have investigated the association between HT and TCs other than PTC and the mechanisms that might explain such associations. Therefore, the number of patients with TCs other than PTC included in our study is also limited compared to patients with PTC. This affects the strength of the statistical analysis regarding these subtypes of TC, primarily MTC and ATC.

In general, several aspects should be considered regarding bias, in particular selection bias. Studies examining associations between HT and TC are very heterogeneous in terms of diagnostic methods for HT challenging valid evaluations, and statistical strength also varies considerably among studies. Furthermore, few studies have available data on when HT was diagnosed, i.e., if patients had HT prior to the diagnosis of TC, or if the diseases were diagnosed simultaneously. This makes it difficult to determine whether patients have HT prior to their cancer or if their cancer might have induced HT. Likewise, time from HT diagnosis to development of TC remains unknown for patients with prior HT.

Our systematic review found an association between HT and PTC among HT and TC patients and between HT and TL among HT patients with RRs ranging from 1.4 to 9.7. We should therefore consider wider indications for a workup when this patient group presents suspect symptoms of cancer. The prognosis of these subtypes of TC is favorable, but there is space for further improvement and even though our study did not find a correlation between HT and subtypes of TC with poor prognosis, i.e., MTC and ATC, studies on this matter are limited and such an association might exist after all. Increased awareness of suspect symptoms of cancer among patients with HT might therefore identify such TCs earlier and thus improve their prognosis as well.

To better understand and validate the association between HT and TC and to exclude biases, prospective studies involving large cohorts and long-term follow-up are needed. Furthermore, uniform diagnostic criteria would strengthen future research.

## Author Contributions

CP: Idea development, data collection and analysis, interpretation of data, manuscript preparation, critical revision of the article, and final approval of the version to be published. CG: Idea development, interpretation of data, manuscript preparation, critical revision of the article, supervision of the development of work, and final approval of the version to be published. CB: Idea development, critical revision of the article, supervision of the development of work, manuscript evaluation, and final approval of the version to be published and acted as corresponding author. UF-R: Critical revision of the article, supervision of the development of work, manuscript evaluation, and final approval of the version to be published.

## Conflict of Interest Statement

The authors declare that the research was conducted in the absence of any commercial or financial relationships that could be construed as a potential conflict of interest.
